# Microvascular remodeling and endothelial dysfunction across the post-COVID-19 spectrum: a prospective observational case-control study

**DOI:** 10.1186/s12916-026-05144-9

**Published:** 2026-08-19

**Authors:** Timon Wallraven, Roman Günthner, Isabelle Lethen, Andrea Ribeiro, Maciej Lech, Frederike Cosima Oertel, Lukas Gabriel Reeß, Bernhard Haller, Lukas Streese, Henner Hanssen, Michael Basta-Wunderle, Christoph Schmaderer

**Affiliations:** 1 TUM School of Medicine and Health, Department of Nephrology, TUM University Hospital, Technical University of Munich, Munich, Germany; 2 Department of Medicine IV, LMU University Hospital, Munich, Germany; 3 Experimental and Clinical Research Center, Center for Molecular Medicine, Charité-Universitätsmedizin Berlin, Corporate Member of Freie Universität Berlin and Humboldt-Universität zu Berlin, Berlin, Germany; 4 Einstein Center Digital Future Berlin, Berlin, Germany; 5 Department of Neurology, Corporate Member of Freie, Max Delbrück Center for Molecular Medicine, Charité-Universitätsmedizin Berlin, Universität Berlin and Humboldt-Universität zu Berlin, Berlin, Germany; 6 TUM School of Medicine and Health, Institute for AI and Informatics in Medicine, TUM University Hospital, Technical University of Munich, Munich, Germany; 7 Faculty of Health Care, Therapeutic Sciences, Niederrhein University of Applied Sciences, Krefeld, Germany; 8 Department of Nephrology, Medical Faculty, University Hospital Düsseldorf, Heinrich-Heine- University Düsseldorf, Düsseldorf, Germany; 9 Department of Sport, Exercise and Health, Preventive Sports Medicine and Systems Physiology, University of Basel, Basel, Switzerland; 10 German Centre for Infection Research (DZIF), Partner Site Munich, Munich, Germany

**Keywords:** Post-COVID-19 syndrome, Myalgic encephalomyelitis/chronic fatigue syndrome, Endothelial dysfunction, Retinal microcirculation, Retinal vessel analysis, Neurovascular coupling

## Abstract

**Background:**

Post-viral diseases, including post-COVID-19 syndrome (PCS) and myalgic encephalomyelitis/chronic fatigue syndrome (ME/CFS), cause substantial long-term morbidity. Persistent cardiovascular (CV) risk after acute infection highlights the need for accessible tools to quantify microvascular health.

**Methods:**

The “All Eyes on PCS” is a prospective observational study assessing the microcirculation using retinal vessel analysis (RVA). Both dynamic and static parameters (DVA and SVA) were compared across never SARS-CoV-2 infected individuals (NI, *n* = 96), SARS-CoV-2 recovered individuals (*n* = 102), and PCS patients (*n* = 102), including a subgroup fulfilling ME/CFS criteria (*n* = 62). Analyses were complemented by propensity score-based weighting and multivariable adjustment. Associations with symptom severity and circulating biomarkers of endothelial dysfunction and inflammation were examined.

**Results:**

PCS patients showed reduced venular flicker-induced dilation compared with recovered individuals (3.7% ± 2.2 vs. 4.8% ± 3.0, *p* = 0.006) and narrower retinal arterioles (CRAE) compared with both recovered (178.3 ± 15.5 μm vs. 186.1 ± 15.7 μm, *p* = 0.001) and NI individuals (184.4 ± 14.3 μm, *p* = 0.009). Arteriolar-to-venular ratio (AVR) was lower in PCS compared with NI (0.83 ± 0.06 vs. 0.87 ± 0.06, *p* < 0.001) and recovered participants (0.86 ± 0.07, *p* = 0.034). Findings remained largely consistent after age and sex balancing and adjustment for CV risk factors, although the association for AVR was attenuated. PCS patients fulfilling ME/CFS criteria showed the most pronounced retinal microvascular alterations, and a combined model discriminated ME/CFS patients with good accuracy (AUC = 0.79). Higher symptom burden was associated with lower AVR (*r* = − 0.21, *p* = 0.037), particularly for neurocognitive symptoms. IL-6, ICAM-1, and VCAM-1 were elevated in PCS and ME/CFS, and lower AVR was associated with inflammatory and iron-related markers (all adjusted *p* < 0.01).

**Conclusions:**

PCS is associated with persistent endothelial dysfunction, most pronounced in ME/CFS patients and linked to symptom severity and ongoing inflammation. These findings support the potential use of RVA as a non-invasive tool for assessing and monitoring endothelial health in post-viral syndromes, with implications for cardiovascular risk stratification.

**Trial Registration:**

The All Eyes on PCS Study has previously been registered at ClinicalTrials.gov (NCT05635552).

**Supplementary Information:**

The online version contains supplementary material available at 10.1186/s12916-026-05144-9.

## Background

Post-COVID-19 syndrome (PCS), also referred to as long COVID, remains a major public health challenge, frequently resulting in prolonged morbidity, work incapacity, and a substantial socioeconomic burden [1–4]. Although multiple terms and definitions are used by different authorities, PCS is commonly defined as the persistence or development of new symptoms three months after the initial onset of SARS-CoV-2 infection, lasting for at least two months and not attributable to alternative diagnoses. While many individuals recover fully from acute infection, a considerable subset experiences persistent, multisystem symptoms, including fatigue, cognitive impairment, and exercise intolerance, that may persist for months to years [5, 6]. A subset of PCS patients fulfills diagnostic criteria for myalgic encephalomyelitis/chronic fatigue syndrome (ME/CFS), characterized by profound fatigue and post-exertional malaise (PEM) [7].

Multiple, potentially interacting mechanisms include viral persistence, immune dysregulation, autoimmunity, metabolic and mitochondrial dysfunction, muscle and neurovascular (NV) alterations, and persistent endothelial dysfunction (ED) [6, 8–11]. While these processes contribute to the marked biological heterogeneity of post-viral syndromes such as PCS and ME/CFS, they may converge on shared downstream alterations at the level of endothelial function [12–14]. Accordingly, circulating biomarkers of ED, including intercellular adhesion molecule-1 (ICAM-1) and vascular cell adhesion molecule-1 (VCAM-1), vascular endothelial growth factor (VEGF), pro-inflammatory chemokines and cytokines including C-C motif chemokine ligand-5 (CCL-5), interleukin-6 (IL-6), monocyte chemoattractant protein-1 (MCP-1), and C-X-C motif chemokine ligand-10 (CXCL-10) have been reported in acute COVID-19, post-COVID-19 syndrome (PCS), and myalgic encephalomyelitis/chronic fatigue syndrome (ME/CFS) [15–19].

SARS-CoV-2 infection confers a sustained increase in cardiovascular (CV) risk extending beyond the acute phase, even after mild infection, supporting the concept of persistent vascular injury and accelerated vascular ageing as a central long-term consequence of COVID-19 [20–22]. Consequently, persistent macrovascular and microvascular dysfunction assessed by flow-mediated dilation, pulse-wave velocity, post-occlusive reactive hyperemia and optical coherence tomography angiography have been reported in patients with ongoing symptoms [14, 22–25].

Notably, neurocognitive symptoms such as fatigue, impaired concentration, and reduced executive function have been linked to NV involvement and disturbed cerebral perfusion in post-viral diseases, suggesting that ED may contribute to central nervous system manifestations [26, 27].

In this context, retinal vessel analysis (RVA), including static (SVA) and dynamic retinal vessel analysis (DVA), may provide a non-invasive window into systemic and cerebral microvascular function [28, 29]. The retinal microvasculature shares embryological origin, anatomical features, and autoregulatory mechanisms with the cerebral microcirculation. Retinal flicker-induced vasodilation (FID) reflects NV coupling mediated by nitric oxide (NO)-dependent endothelial signaling [30, 31]. DVA non-invasively quantifies FID of retinal arterioles (aFID) and venules (vFID) as the percentage change from baseline diameter in response to flickering light stimulation and thereby serves as a surrogate of NV coupling and endothelial function. In a cohort of patients undergoing dialysis, we previously demonstrated that reduced vFID is an independent predictor of all-cause mortality [32]. Retinal FID correlates with dynamic cerebral hemodynamic changes assessed by functional MRI, highlighting DVA as a non-invasive approach to probe NV function relevant to cognition [33].

SVA predominantly captures structural microvascular remodeling. Retinal vessel calibers, including the central retinal arteriolar equivalent (CRAE) and central retinal venular equivalent (CRVE), reflect microvascular structural integrity. The arteriolar-to-venular ratio (AVR) represents a dimensionless ratio, which reflects the relative relationship between arteriolar narrowing and venular widening and thereby serves as an integrated marker of microvascular remodeling [34]. Changes in SVA parameters reflect systemic vascular health and have been validated as predictors of CV outcomes in large cohort studies [35–37].

Despite growing evidence for objective biological correlates of persistent post-viral symptoms, accessible non-invasive tools to quantify microvascular dysfunction remain limited, creating a gap in clinical assessment and monitoring [6, 16]. As a result, comprehensive comparative studies comparing endothelial function across never infected (NI) controls, SARS-CoV-2 recovered individuals, PCS patients, and ME/CFS patients are lacking. It remains unclear whether the degree of ED correlates with symptom severity or inflammatory status in PCS.

The primary aim of this study was to characterize retinal microvascular alterations across the post-infectious spectrum, comparing NI, recovered, and PCS individuals, including a subgroup fulfilling ME/CFS criteria. Secondary objectives included the assessment of associations between RVA parameters, patient-reported outcome measures (PROMs), and circulating markers of ED and inflammation.

## Methods

### Study design and cohort

This study is based on the previously published “All Eyes on PCS” protocol, a prospective, observational, single-center study designed to examine the retinal microvasculature in PCS patients [38]. The study protocol was approved by the Ethics Committee of the Technical University of Munich, TUM School of Medicine and Health, Klinikum Rechts der Isar (Approval number: 2022-317-S-SR) and is registered at ClinicalTrials.gov (NCT05635552). The study was conducted in accordance with the Declaration of Helsinki. Sample size was defined a priori in the study protocol based on power calculations conducted with the institute of Artificial Intelligence and Informatics in Medicine (TUM School of Medicine and Health).

Between October 2022 and September 2023, 105 PCS patients meeting the WHO definition of post-COVID-19 condition (persistent symptoms ≥ 3 months after confirmed SARS-CoV-2 infection, not explained by an alternative diagnosis) were included in the study [5]. Most participants (72.4%, 76/105) were recruited through social media with the support of the patient organization “lost voices”, while the remaining (27.6%, 29/105) were referred from the PCS outpatient clinic of the LMU University Hospital Munich (LMU). Predefined exclusion criteria were missing or incomplete consent, age < 18 years, pregnancy, malignancy, diseases associated with reduced life expectancy, autoimmune/rheumatological diseases, cataract, epilepsy, and glaucoma. Three patients were excluded from the analysis: one due to insufficient evidence of SARS-CoV-2 infection, another because of a missing temporal link between infection and PCS symptom onset, and a third because PCS-related symptoms were absent at the time of recruitment. In addition, 105 SARS-CoV-2–recovered individuals without persistent symptoms were recruited as controls and examined between October 2022 and January 2025. Three individuals were excluded from the final analysis: one due to a diagnosis of myocarditis, one due to epilepsy precluding RVA, and one due to incomplete data. NI participants were selected from a cohort of 233 individuals with no history of prior SARS-CoV-2 infection. Of these, 227 were recruited before the COVID-19 pandemic as control participants as part of the Citrate-Acetate Study (recruited between 2016 and 2018; ClinicalTrials.gov: NCT02745340) and the COMPLETE Study (recruited between January 2018 and July 2019; ClinicalTrials.gov: NCT03986892) [28]. Six control participants were recruited after the COVID-19 pandemic who reported no prior SARS-CoV-2 infection. A flowchart on participant selection, exclusions, and analysis strategies is provided in Additional File 1: Fig S1.

### Clinical assessment

A standardized 78-item questionnaire (Munich COVID History Questionnaire; MuCOV) was used to collect demographic data, comorbidities, medication history, and PCS-related symptoms. All participants underwent a structured physician-led clinical assessment. Participants recruited via social media completed an initial screening survey capturing acute SARS-CoV-2 infection characteristics and persistent symptoms. Eligibility for both PCS and recovered groups required documented SARS-CoV-2 infection ≥ 3 months prior to study inclusion. PCS was defined by the presence of typical symptoms persisting for ≥ 2 months after acute infection. The temporal relationship between infection and symptom onset was verified, and alternative diagnoses were systematically excluded. In cases of diagnostic uncertainty, eligibility was adjudicated in multidisciplinary meetings, with final decisions made by majority vote. PROMs were assessed: PCS Severity (PCS Severity Score [39] and COVID-19 Yorkshire Rehabilitation Scale (C19-YRS) [40]), fatigue (Fatigue Severity Scale, FSS [41]), depression (Patient Health Questionnaire-9, PHQ-9 [42]), anxiety disorders (Generalized Anxiety Disorder-7, GAD-7 [43]), health-related quality of life (EuroQol 5-Dimensions 5-Levels questionnaire (EQ-5D-5 L [44]) and ME/CFS status using the Canadian Consensus Criteria (CCC) [45].

### Laboratory values

Blood sampling and standard laboratory measurements were conducted as previously described [46], with IL-6, CCL-5, CXCL-10, MCP-1, VCAM-1, ICAM-1 and VEGF quantified using the Cytometric Bead Array Flex system (BD Biosciences, San Diego, US). The measurements were performed according to the manufacturer’s instructions.

### Retinal vessel analysis

RVA was conducted with the Dynamic Vessel Analyzer (DVAlight; Imedos Health GmbH, Jena, Germany), while static vessel analysis (SVA) was carried out using the Static Vessel Analyzer (Imedos Health GmbH, Jena, Germany) in combination with a TRC-NW8 non-mydriatic retinal camera (Topcon, Tokyo, Japan). A detailed description of the procedure can be found in a previous publication [46].

All measurements were performed in a quiet, temperature-controlled room. Blood pressure was assessed before and after RVA. Pupillary dilation was induced with topical tropicamide. Whenever feasible, the left eye was examined, and the contralateral eye was occluded. For SVA, ≥ 3 high-quality fundus images centered on the optic disc were acquired. Retinal vessel calibers were quantified semi-automatically using VesselMap (Imedos Health GmbH, Jena, Germany) and summarized as CRAE and CRVE according to the Parr-Hubbard formula. AVR was calculated as CRAE/CRVE. One measuring unit corresponds to 1 μm (Gullstrand model). Images of insufficient quality were excluded (1/102 PCS; 1.0%).

For DVA, vessel segments (around 0.5 to 1 mm in length) were selected approximately two optic-disc diameters from the disc margin, preferentially in the superior-temporal quadrant. Vessel diameters were continuously recorded over 350 s, including a baseline period and three flicker cycles (20 s each) with recovery phases. FID was calculated as percentage change from baseline (aFID, vFID). RVA acquisition was performed by trained personnel not blinded to clinical group. All RVA data processing, quality control, and quantitative analyses were conducted by investigators blinded to clinical group assignment. The IMEDOS-derived Valid% was 78.6% (IQR 68.1 to 85.5%) for vFID recordings and 74.5% (IQR 65.5 to 84.5%) for aFID in our cohort. To ensure high measurement quality, vessel response curves were rated using a cumulative scoring approach (scale 0–5), incorporating evaluation of flicker response interpretability, presence of at least one evaluable flicker cycle, consistency of measuring points, signal noise, and recording gaps/artifacts as previously described [47]. Recordings with a total score < 2.5 were re-evaluated by a second experienced observer and excluded if consensus could not be reached [47]. Quality scores were consistently high across cohorts: vFID QC score: NI, 4.5 (3.5 to 5.0), recovered, 4.0 (4.0 to 4.5), PCS, 4.0 (4.0 to 4.5); aFID QC score: NI, 4.25 (3.0 to 4.5), recovered, 4.5 (4.0–5.0), PCS, 4.5 (4.0–5.0).

### Data analysis

All statistical analyses were performed using R (version 4.2.1; R Foundation for Statistical Computing, Vienna, Austria) in RStudio and followed the “Strengthening the Reporting of Observational studies in Epidemiology” (STROBE) guidelines. Analyses were conducted using complete-case data. No imputation was performed, as missingness was low and did not show systematic patterns. Normality was assessed by visual inspection of histograms and Q-Q plots and formally tested using the Shapiro-Wilk test. Normally distributed variables are presented as mean ± standard deviation (SD), and non-normally distributed variables as median with interquartile range (Q1, Q3). For analyses, participants were categorized into PCS, recovered, and NI groups. Due to age differences and limited overlap between PCS, NI and recovered participants, matching strategies were applied. For comparisons between PCS and NI, an age- and sex-matched NI cohort was derived using nearest-neighbor matching, with balance assessed using the MatchBalance function. Comparisons between PCS and recovered participants were performed using propensity score-based overlap weighting targeting the average treatment effect in the overlap population (ATO). We applied overlap weighting using the WeightIt package to balance age and sex between groups. Covariate balance was excellent (all standardized mean differences < 0.1; Additional File 1: Table S1). Weighted comparisons of RVA and laboratory parameters were conducted using survey-weighted linear models implemented in the survey package. In addition, we performed a strict exact age- and sex-matched subgroup analysis between recovered and PCS participants using nearest-neighbor matching. One eye per participant was included in the analysis. Unweighted group comparisons were conducted using Welch’s two-sample t-test or the Mann-Whitney U test for two-group comparisons and the Chi-square (χ^2^) test for categorical variables. For comparisons involving more than two groups, we used one-way ANOVA when model assumptions were met; otherwise, the Kruskal-Wallis test was applied. Following significant ANOVA results, post-hoc pairwise comparisons were performed using pairwise t-tests with Hochberg adjustment for multiple testing. For Kruskal-Wallis, post-hoc pairwise comparisons were performed using Dunn’s test with multiplicity correction. Additionally, multivariable linear regression models were fitted to adjust for age and established confounders of RVA parameters [48–51]. Continuous biomarkers with skewed distributions or outliers were analyzed using robust linear regression based on M-estimation. Correlation analyses were performed using Pearson’s correlation for normally distributed data and Spearman’s rank correlation for non-normally distributed data. For correlation analyses involving multiple biomarkers, p-values were adjusted using the Benjamini-Hochberg procedure. Receiver operating characteristic (ROC) analyses were used to assess the discriminative ability of individual variables, with confidence intervals calculated using DeLong’s method. To evaluate whether a combination of inflammatory, metabolic, and microvascular markers improves discrimination of the ME/CFS phenotype among SARS-CoV-2-infected individuals, a multivariable logistic regression model including transferrin, ICAM-1, neutrophils, creatine kinase, IL-6 and AVR was fitted (Additional File 1: Table S2). ME/CFS status served as the dependent variable. Predicted probabilities were used to construct ROC curves and estimate the area under the curve (AUC).

## Results

### Cohort characteristics across PCS patients, recovered, and never infected individuals

Summarizes the baseline characteristics of the three study groups: NI controls matched for age and sex (*n* = 96), recovered individuals (*n* = 102) and PCS patients (*n* = 102).Age differed significantly between groups, with recovered individuals being younger (33.3 ± 11.4 years, 72% female) than both NI (43.0 ± 14.5 years, 76% female) and PCS patients (41.9 ± 11.6 years, 75% female). Systolic blood pressure before RVA varied between cohorts, with recovered participants showing the lowest values. CV risk such as arterial hypertension was present in 16% of PCS patients compared with 4.9% of recovered and 0% of NI individuals, and hypercholesterolemia was more common in PCS than in recovered (22% vs. 5.9%). Acute SARS-CoV-2 infection characteristics showed differences: PCS patients experienced more severe acute disease, while most recovered and PCS individuals reported a single infection, and time since first acute infection was comparable between groups. PCS burden remained high, with a median symptom duration of 404 days (IQR: 291 to 575), median cumulative sick leave of 199.5 days (44.0 to 377.0), and 16% reporting occupational loss. PCS symptom severity was markedly elevated compared to recovered (36.9 ± 10.6 vs. 1.8 ± 5.9), and 61% of PCS patients fulfilled ME/CFS diagnostic criteria. Laboratory differences were observed between groups, with PCS patients showing higher inflammatory markers and alterations in iron metabolism compared with recovered and NI individuals. However, most laboratory differences were attenuated after adjustment for age and sex (Additional File 1: Table S3)

**Table 1:** Presents baseline demographic, clinical, and laboratory characteristics of the three study cohorts: Never infected (NI) controls, recovered individuals, and patients with post-COVID-19 Syndrome (PCS)

	NI *n* = 96	Recovered *n* = 102	PCS *n* = 102	*p*-value
**Baseline characteristics**				
Age, years, mean (± SD)	43.0 (± 14.5)	33.3 (± 11.4)	41.9 (± 11.6)	<0.001***
Sex, female, n (%)	73 (76%)	73 (72%)	77 (75%)	>0.9
BMI, kg/m^2^, mean (± SD)	23.2 (± 3.7)	23.5 (± 4.0)	24.5 (± 4.8)	0.090
Resting systolic BP before RVA, mmHg, mean (± SD)	122.7 (± 16.0)	116.7 (± 15.7)	121.8 (± 16.9)	0.026*
**Cardiovascular Risk Factors**				
Arterial Hypertension, n (%)	0 (0%)	5 (4.9%)	16 (16%)	<0.001***
Hypercholesterolemia, n (%)	-	6 (5.9%)	22 (22%)	0.003**
Nicotine abuse, n (%)	11(11%)	14 (14%)	10 (9.8%)	0.70
Diabetes mellitus, n (%)	0 (0%)	0 (0%)	1 (1.0%)	0.40
Acute SARS-CoV-2 Infection Characteristics				
Days since acute infection	-	486.0 (276.0, 736.0)	405.0 (291.0, 575.0)	0.20
Number of infections, n (%)				-
0	96 (100%)	0 (0%)	0 (0%)	
1	0 (0%)	62 (61%)	71 (70%)	
≥2	0 (0%)	40 (39%)	31 (30%)	
Severity of acute infection, n (%) (WHO progression scale)				<0.001***
WHO scale 1 and 2	-	10 (9.8%)	1 (1.0%)	
WHO scale 3 and 4	-	91 (89.2%)	95 (93.1%)	
WHO scale ≥ 5	-	1 (1.0%)	6 (5.9%)	
**PCS** ***-*** **related Characteristics**				
Duration of PCS, days, median (IQR)	0.0 (0.0, 0.0)	0.0 (0.0, 0.0)	404.0 (291.0, 575.0)	-
Cumulative sick days, median (IQR)	-	0.0 (0.0, 0.0)	199.5 (44.0, 377.0)	<0.001***
Work loss during PCS, n (%)	-	-	16 (16%)	**-**
PCS Severity Score, mean (± SD)	-	1.8 (5.9)	36.9 (10.6)	<0.001***
C19-YRS, mean (± SD)	-	-	32.4 (14.0)	-
FSS, median (IQR)	-	2.3 (1.6, 3.1)	6.1 (5.1, 6.7)	<0.001***
ME/CFS, n (%)	-	0 (0%)	62 (61%)	-
**Comorbidities**				
Depression, n (%)	-	10 (9.8%)	19 (19%)	0.20
Pre-existing mental conditions, n (%)	-	4 (4.0%)	8 (7.9%)	0.40
**Laboratory Values**				
Leukocytes (G/L), mean (± SD)	6.0 (±1.5)	6.5 (±1.8)	6.7 (±1.8)	0.063
CRP (mg/dl), median (IQR)	0.05 (0.02, 0.09)	0.10 (0.10, 0.20)	0.09 (0.09, 0.20)	<0.001***
Neutrophils (%), mean (± SD)	-	58.2 (±9.4)	62.5 (±8.4)	0.001**
Lymphocytes (%), mean (± SD)	-	30.5 (±9.0)	28.0 (±7.7)	0.041*
Hemoglobin (g/dL), mean (± SD)	14.5 (±1.5)	13.4 (±1.6)	13.8 (±1.3)	<0.001***
Ferritin (µg/L), median (IQR)	-	58.5 (27.5, 106.0)	78.0 (50.0, 152.0)	0.008**
Transferrin (mg/dL), mean (± SD)	-	286.0 (±46.2)	260.9 (±33.2)	<0.001***

Continuous variables are reported as mean (± SD) or median (Q1, Q3), depending on distribution, and categorical variables as n (%). Statistical comparisons across groups were performed using one-way ANOVA, the Kruskal-Wallis test, or Pearson’s χ^2^ test, as appropriate. Abbreviations: Never infected, NI; BMI, Body Mass Index; C19-YRS, COVID-19 Yorkshire Rehabilitation Scale; FSS, Fatigue Severity Scale; ME/CFS, Myalgic Encephalomyelitis/Chronic Fatigue Syndrome; Missing data are reported as number of missing observations (n) for each group in the following order: NI, recovered, and PCS: systolic blood pressure before RVA [2, 7, 11]; hypercholesterolemia (-, 0, 0); work loss during PCS (-, 0, 2); PCS Severity Score (-, 3, 0); C19-YRS score (-, 102, 1); Fatigue Severity Scale (-, 5, 1); ME/CFS status (-, 0, 1); depression diagnosis (-, 0, 0); pre-existing mental conditions (-, 1, 1); leukocytes [4, 8, 50]; CRP [3, 7, 62]; neutrophils (-, 12, 7); lymphocytes (-, 8, 4); hemoglobin [4, 7, 52]; ferritin (-, 6, 5); transferrin (-, 6, 5); Statistical significance is displayed as: * *p* < 0.05; ** *p* < 0.01;*** *p* < 0.001

### Functional and structural retinal microvascular alterations across PCS, recovered, and never infected

**Fig. 1:**
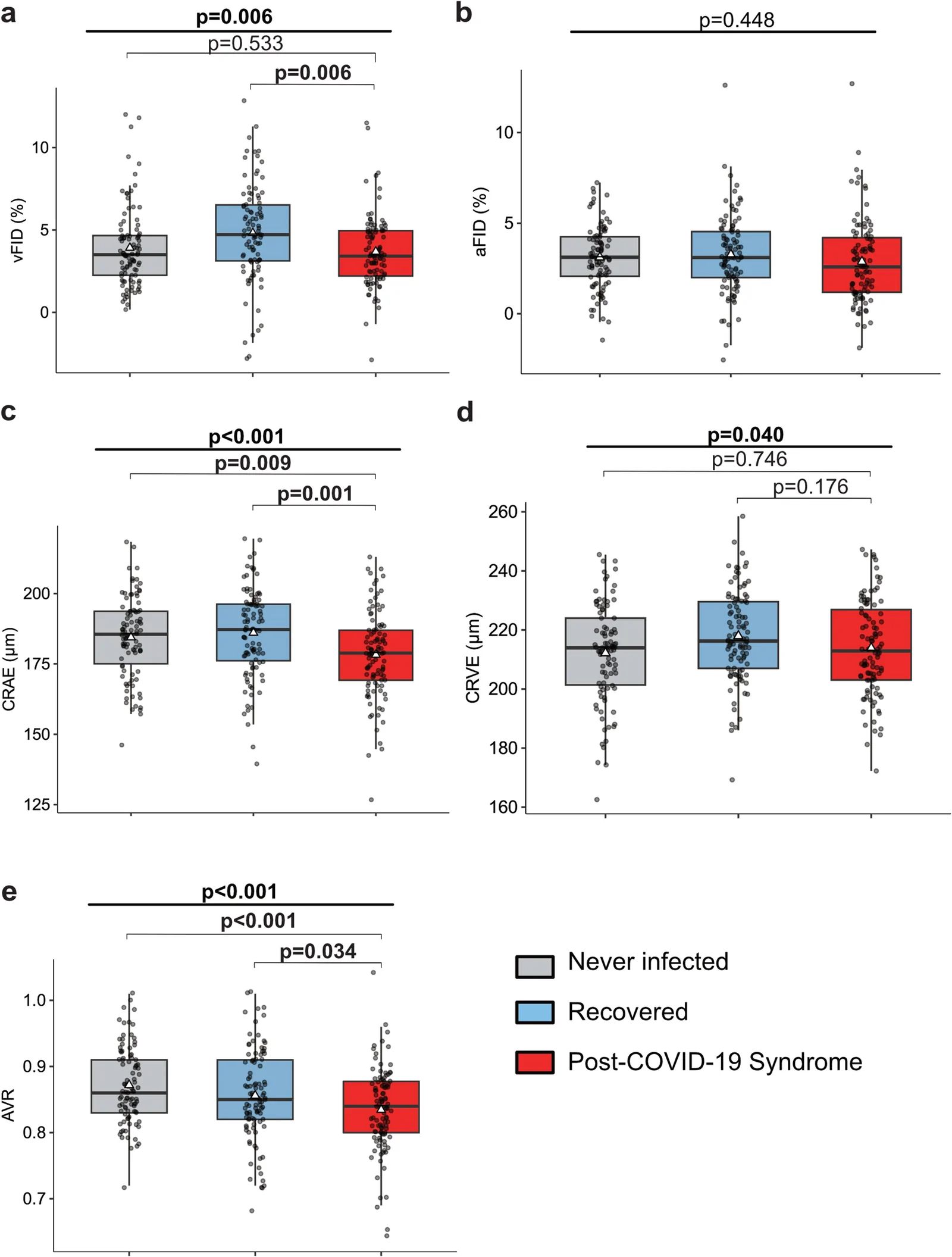
Retinal microvascular parameters across never infected, recovered, and PCS cohorts. Box plots show DVA parameters vFID (NI *n* = 96; recovered *n* = 95; PCS *n* = 99; **a**) and aFID (NI *n* = 95; recovered *n* = 97; PCS *n* = 99; **b**), and SVA parameters CRAE (**c**), CRVE (**d**), and AVR (NI *n* = 94; recovered *n* = 99; PCS *n* = 102; **e**). Groups are visualized as never infected (grey), recovered (light blue), and PCS (red). Box plots indicate the median (horizontal line) and mean (black dot). Each data point represents one eye from one individual (one eye analyzed per participant). Group comparisons were performed using one-way ANOVA for normally distributed variables. Post-hoc pairwise comparisons were performed using pairwise t-tests with Hochberg adjustment for multiple testing

DVA parameters varied across NI, recovered, and PCS participants (Fig. 1). Mean venular FID (vFID) differed between the three groups (*p* = 0.006). In post-hoc comparisons, PCS patients showed lower vFID than recovered individuals (3.7% ± 2.2 vs. 4.8% ± 3.0, *p* = 0.006). No differences were observed between PCS and NI (3.9% ± 2.4, *p* = 0.533). Arteriolar FID showed a trend toward lower values in PCS compared with recovered individuals (2.9% ± 2.3 vs. 3.3% ± 2.2), but this did not reach significance (Fig. 1a and b). SVA parameters also showed significant between-group differences. Retinal arteriolar diameters (CRAE) differed (*p* < 0.001). PCS patients had narrower arterioles compared to both recovered (178.3 ± 15.5 μm vs. 186.1 ± 15.7 μm, *p* = 0.001) and NI (184.4 ± 14.3 μm, *p* = 0.009). Retinal venular diameters showed modest between-group differences and did not differ significantly between PCS patients and either NI controls (*p* = 0.746) or recovered individuals (*p* = 0.176). (Fig. 1c and d). AVR varied between groups (*p* < 0.001). PCS patients exhibited lower AVR compared with both NI (0.83 ± 0.06 vs. 0.87 ± 0.06, *p* < 0.001) and recovered (0.86 ± 0.07, *p* = 0.034). (Fig. 1e) Given the younger age of the recovered cohort, we performed an age- and sex-balanced overlap-weighted sensitivity analysis based on propensity scores (ATO). The overall pattern remained consistent, confirming lower venular FID, as well as narrower CRAE, in PCS compared with recovered individuals. The difference in AVR was attenuated and narrowly missed statistical significance (*p* = 0.056), whereas CRVE did not differ significantly between groups. (Additional File 1: Table S4). Second, in a subgroup individually matched 1:1 for age and sex (mean age, 36.6 years in both groups), the key findings were reproduced. Participants with PCS exhibited lower vFID (3.5 ± 2.1% vs. 4.9 ± 2.9%, *p* = 0.010), narrower CRAE (180.7 ± 13.1 μm vs. 188.6 ± 14.3 μm, *p* = 0.008), and a lower AVR (0.83 ± 0.05 vs. 0.87 ± 0.07, *p* = 0.007) than recovered participants. CRVE remained unchanged (217.1 ± 15.8 μm vs. 218.1 ± 14.3 μm, *p* = 0.762). The difference in aFID was directionally similar but did not reach statistical significance (2.7 ± 2.2% vs. 3.3 ± 2.6%, *p* = 0.232; Additional File 1: Table S5). Finally, multivariable regression analyses adjusting for age and CV risk factors further confirmed these findings. Compared with PCS, higher vFID remained independently associated with recovered status (Std. β = 0.24, *p* = 0.001), whereas no significant association was observed for NI (Std. β = 0.05, *p* = 0.504). Higher CRAE and AVR remained independently associated with NI.

status (CRAE: Std. β = 0.17, *p* = 0.010; AVR: Std. β = 0.23, *p* = 0.001), while weaker non-significant associations were observed for recovered individuals (CRAE: Std. β = 0.10, *p* = 0.130; AVR: Std. β = 0.10, *p* = 0.139; Additional File 1, Table S6).

### Association of patient-reported outcome measurements with persistent changes of retinal microcirculation

**Fig. 2:**
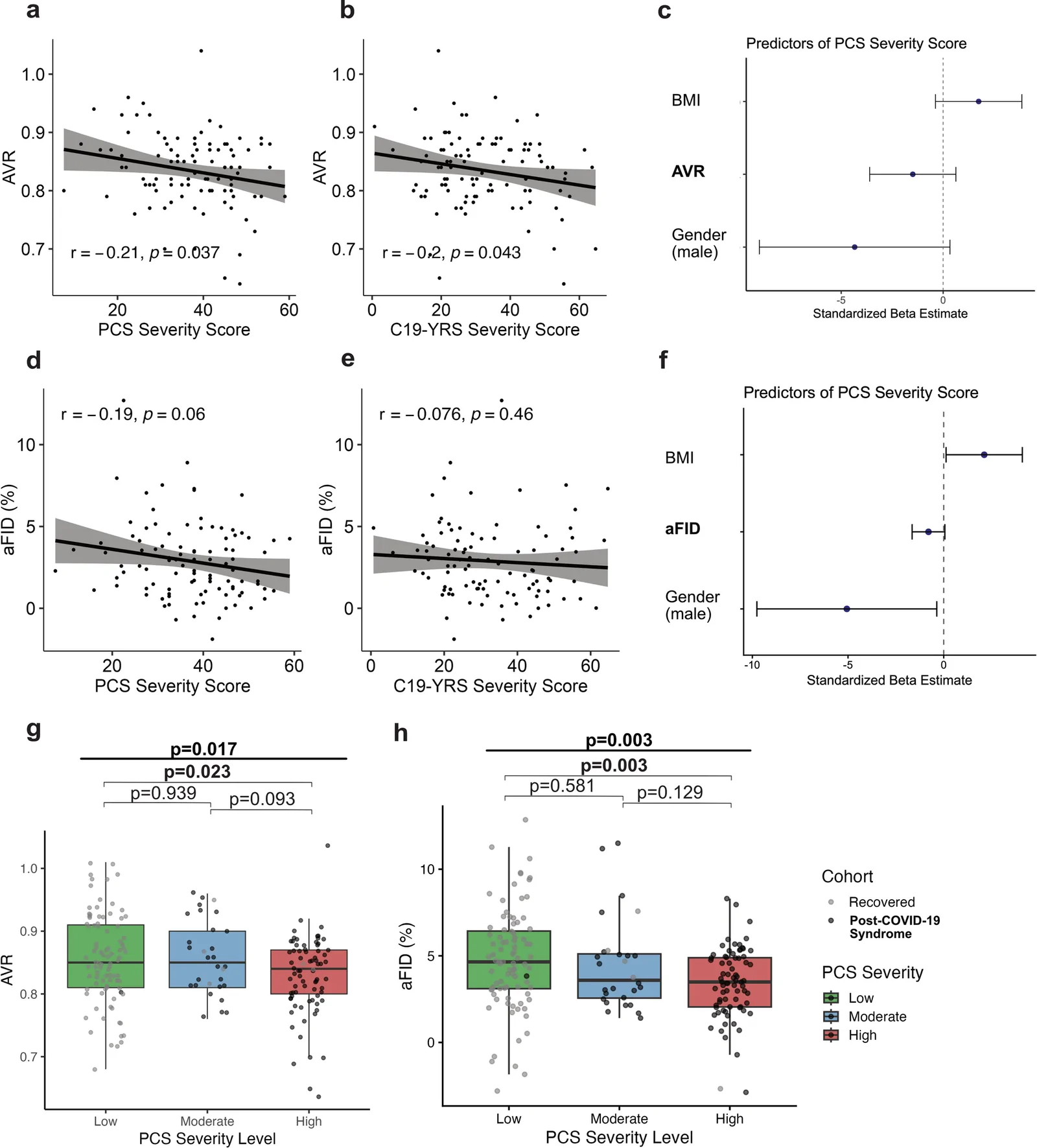
Pearson correlations between RVA parameters and symptom scores in PCS patients. Panels **a-b** show correlations between AVR and the PCS Severity Score (**a**) and C19-YRS (**b**). Panels **d-e** show corresponding correlations for aFID with the PCS Severity Score (**d**) and C19-YRS (**e**). Points represent individual patients; the solid line indicates the linear regression fit and the shaded band the 95% confidence interval. Pearson correlation coefficients (r) and p-values are shown in each panel. **(c**,** f)** Forest plots display standardized β-coefficients with 95% confidence intervals from multivariable regression models assessing the association of AVR (**c**) or aFID (**f**) with the PCS Severity Score after adjustment for BMI and sex. Statistical significance is indicated as *p < 0.05. **(g–h)** Box plots show the distribution of AVR (**g**) and aFID (**h**) across three symptom severity groups in PCS patients (black) and recovered individuals (grey). The PCS Severity Score was categorized into low (green), moderate (blue), and high (red) severity using fixed cutoffs (< 10, 10–30, > 30; colors as indicated). Box plots indicate the median (horizontal line) and individual observations. Group comparisons were performed using one-way ANOVA for normally distributed variables. Post-hoc pairwise comparisons were performed using pairwise t-tests with Hochberg adjustment for multiple testing. Each data point represents one eye from one individual (one eye analyzed per participant)

PROMs, including the PCS Severity Score and the COVID-19 Yorkshire Rehabilitation Scale (C19-YRS), were associated with persistent alterations in retinal microcirculation. AVR showed modest inverse correlations with both the PCS Severity Score (*r* = − 0.21, *p* = 0.037) and C19-YRS scores (*r* = − 0.20, *p* = 0.043; Fig. 2a, b). aFID showed an inverse correlation with the PCS Severity Score that did not reach significance (*r* = − 0.19, *p* = 0.059; Fig. 2d), whereas no correlation was observed with the C19-YRS score (*r* = − 0.08, *p* = 0.460; Fig. 2e). In regression analyses adjusted for sex and BMI, the inverse association between aFID and PCS severity remained directionally consistent (*p* = 0.068), while the association between AVR and PCS severity was attenuated (*p* = 0.165; Fig. 2c, f). Other RVA parameters showed no significant associations with PCS severity within the PCS cohort. To further assess the relationship between symptom burden and RVA parameters, previously infected participants (recovered and PCS) were stratified into tertiles according to their PCS Severity Score. Higher symptom burden was associated with lower AVR (overall *p* = 0.017; high vs. low tertile, *p* = 0.023) and lower aFID (overall *p* = 0.003; high vs. low tertile, *p* = 0.003; Fig. 2g, h). No significant differences were observed between the low and moderate or between the moderate and high tertiles after adjustment for multiple comparisons. In symptom-specific analyses, lower AVR was primarily observed in participants with PCS reporting neurocognitive and general symptoms, whereas no consistent differences were observed for respiratory or sensory symptom domains (Additional File 1, Table S7).

### Retinal microvascular alterations in ME/CFS

**Fig. 3:**
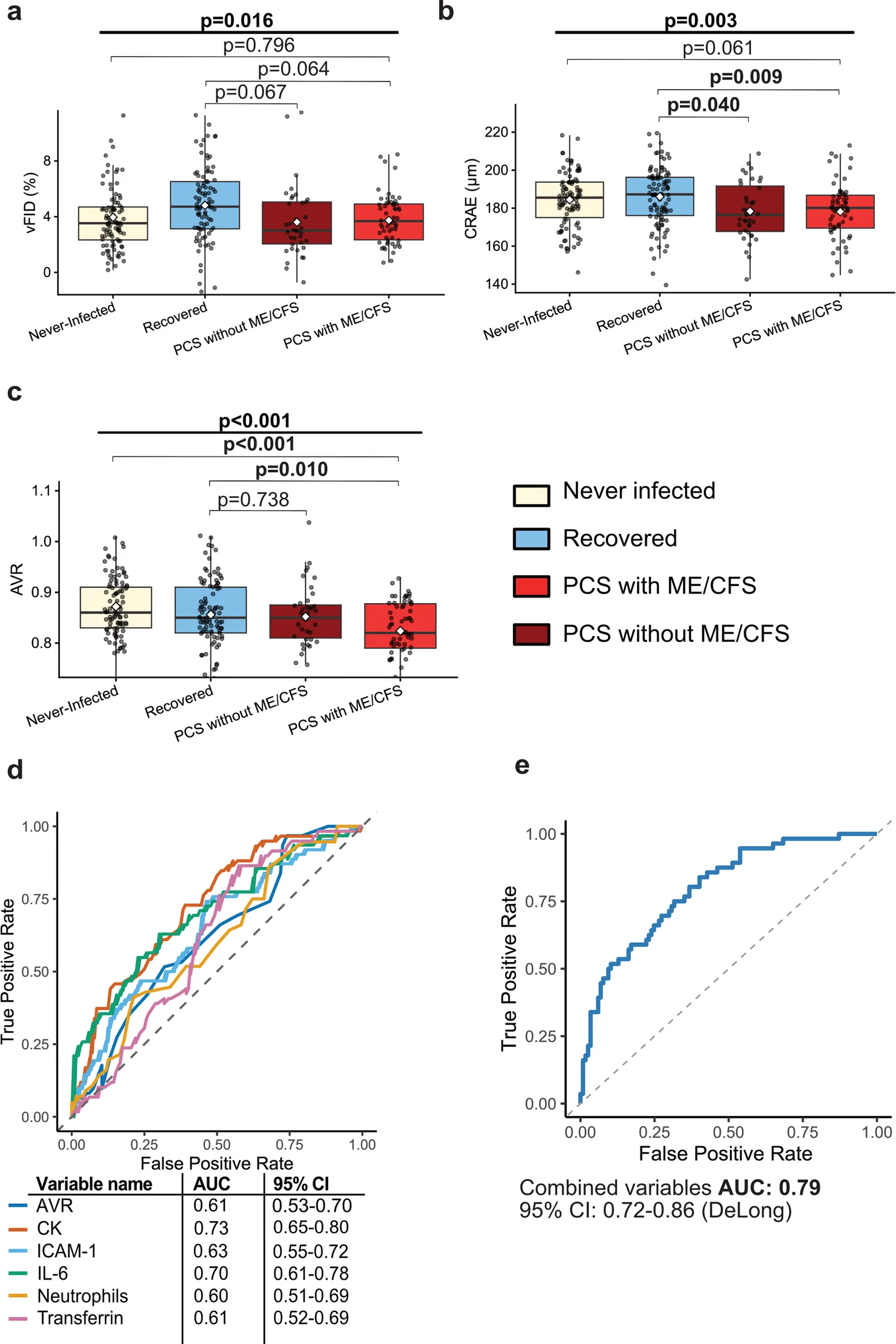
Box plots of the DVA parameter vFID (**a**) and SVA parameters CRAE (**b**) and AVR (**c**) across four cohorts: never infected (*n* = 96, cornsilk), PCS with ME/CFS (*n* = 62, red), PCS without ME/CFS (*n* = 39, dark red), and recovered individuals (*n* = 102, light blue). Box plots indicate the median (horizontal line) and mean (black dot). Group comparisons were performed using one-way ANOVA for normally distributed variables. Post-hoc pairwise comparisons were performed using pairwise t-tests with Hochberg adjustment for multiple testing. Each data point represents one eye from one individual (one eye analyzed per participant). **d** Receiver operating characteristic (ROC) curves for individual biomarkers discriminating PCS patients fulfilling ME/CFS criteria from recovered individuals and PCS patients without ME/CFS. Variables include AVR (dark blue), creatine kinase (CK; red), ICAM-1 (light blue), interleukin-6 (IL-6; green), neutrophils (orange), and transferrin (purple). **e** ROC curve showing discrimination between ME/CFS patients and the combined comparator group (recovered individuals and PCS patients without ME/CFS) using a multivariable model integrating the six variables. Area under the curve (AUC) with 95% confidence interval (CI) was calculated using DeLong’s method

Persistent ED has previously been demonstrated in post-viral syndromes and in patients with ME/CFS [14]. In our cohort PCS patients fulfilling ME/CFS criteria were slightly younger and exhibited a substantially higher symptom burden and reduced quality of life compared with PCS patients without ME/CFS. CV risk factors, acute infection characteristics, and most laboratory parameters were comparable between groups, except for lower creatine kinase (CK) levels in the ME/CFS subgroup (Additional File 1, Table S8). We next compared RVA parameters across NI, recovered, and PCS patients with or without ME/CFS. Post-hoc analyses showed no significant pairwise differences in vFID between the subgroups. Compared with recovered individuals, vFID tended to be lower in participants with PCS with ME/CFS (*p* = 0.064). No differences were observed between either PCS subgroup and NI controls. For SVA parameters, CRAE was lower in PCS patients with ME/CFS than in recovered individuals (*p* = 0.009) and showed a similar trend compared with NI controls (*p* = 0.061). PCS patients without ME/CFS also exhibited lower CRAE than recovered individuals (*p* = 0.040). AVR was lower in PCS patients with ME/CFS than in both NI controls (*p* < 0.001) and recovered individuals (*p* = 0.010). No other pairwise comparisons reached statistical significance (Fig. 3a–c).

In multivariable regression analyses adjusted for age, sex, BMI, systolic blood pressure before RVA, and arterial hypertension, vFID was higher in recovered individuals compared with PCS with ME/CFS (Std. β = 0.22, *p* = 0.006), while no other between-group differences were observed. After adjustment, CRAE differed significantly between groups. Compared with PCS patients with ME/CFS, CRAE was higher in NI individuals (Std. β = 0.19, *p* = 0.013), whereas no significant difference was observed for recovered participants (Std. β = 0.12, *p* = 0.114). AVR was higher in NI (Std. β = 0.31, *p* < 0.001) and recovered individuals (Std. β = 0.17, *p* = 0.029) compared with PCS patients with ME/CFS, while the difference for PCS patients without ME/CFS did not reach statistical significance (Std. β = 0.14, *p* = 0.052) (Additional File 1, Table S9).

Finally, in exploratory analyses, ROC analyses were performed to identify biomarkers discriminating non-ME/CFS individuals (recovered and PCS without ME/CFS) from patients fulfilling ME/CFS criteria. Across multiple biological domains, several markers demonstrated discriminatory performance (Additional File 1, Table S10). From these, six markers (CK, IL-6, ICAM-1, transferrin, AVR, and neutrophils) representing distinct pathophysiological domains were selected for combined ROC analysis, yielding improved discrimination (AUC 0.79, 95% CI 0.72 to 0.86; Fig. 3d**–**e).

### Endothelial and inflammatory biomarkers in recovered, PCS and ME/CFS patients and their association with RVA parameters

**Fig. 4:**
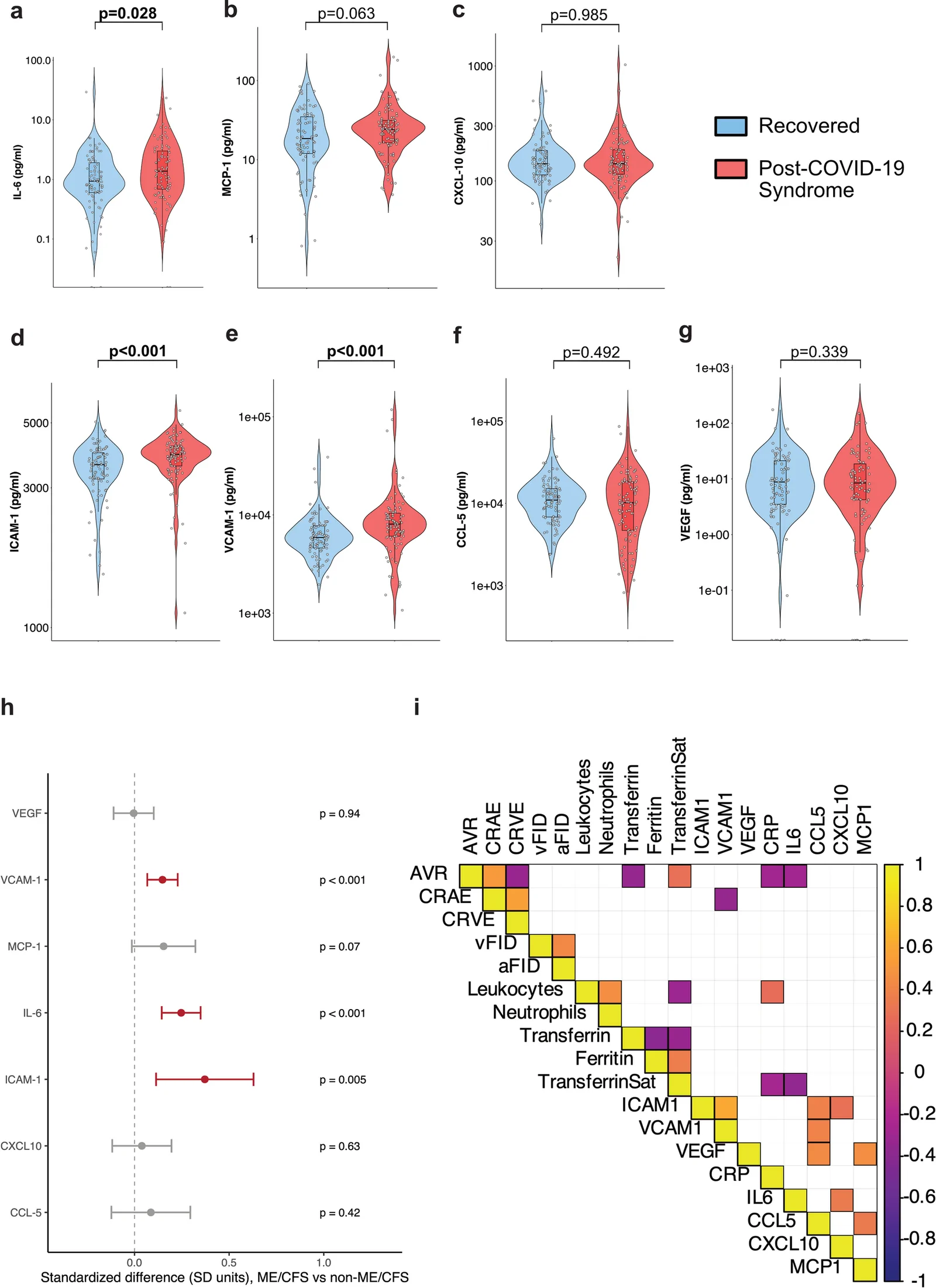
**a**-**g** Violin plots illustrate circulating levels of inflammatory markers (IL-6, MCP-1, CXCL-10, CCL-5) and endothelial dysfunction markers (ICAM-1, VCAM-1, VEGF) in recovered individuals (light blue) and patients with PCS (red). Individual data points represent single participants. Box plots indicate the median (black line) and interquartile range (IQR). Group comparisons were performed using the Wilcoxon rank-sum test for non-normally distributed variables and Welch’s two-sample t-test for normally distributed variables, as appropriate. IL-6, CXCL-10, MCP-1, ICAM-1, VCAM-1, CCL-5, and VEGF were measured in *n* = 90 recovered individuals and *n* = 100 PCS patients. **h** Forest plot illustrating age-adjusted associations between ME/CFS status and seven circulating biomarkers. Effect sizes are presented as standardized differences (SD units) with 95% confidence intervals derived from regression models adjusted for age. Statistically significant associations are highlighted in red (as indicated, *p* < 0.05). **i** Correlation heatmap showing significant Spearman correlations (*p* < 0.05) after Benjamini-Hochberg correction between selected laboratory parameters and RVA parameters AVR, CRAE, CRVE, vFID, and aFID in PCS patients. Spearman’s ρ is color-coded from + 1 (yellow, positive correlation) to − 1 (blue, negative correlation)

Circulating biomarker levels were compared between recovered and PCS patients, to study potential ongoing endothelial activation and chronic inflammation. IL-6 levels were higher in PCS patients compared with recovered individuals (median 1.32 pg/mL vs. 0.92 pg/mL; *p* = 0.028). MCP-1 showed a non-significant trend toward higher levels in PCS (median 24.2 pg/mL vs. 18.5 pg/mL; *p* = 0.063). CC- motif Chemokine-Ligand-5 (CCL-5) and CXCL-10 did not differ between groups (Fig. 4a**–**c, f). Among ED markers, ICAM-1 (mean 3832 ± 636 pg/mL vs. 3505 ± 683 pg/mL, *p* < 0.001) and VCAM-1 (median 8073 pg/mL vs. 5899 pg/mL; *p* < 0.001) levels were elevated in PCS patients. VEGF concentrations did not differ between groups (Fig. 4d, e and g). In ATO-weighted analyses adjusting for age and sex, ICAM-1 and VCAM-1 levels remained significantly higher in PCS compared with recovered individuals, whereas no significant differences were observed for IL-6 and MCP-1 (Additional File 1, Table S11). Laboratory variables and circulating biomarkers were further compared between non-ME/CFS (recovered and PCS without ME/CFS) and ME/CFS patients after age adjustment. Markers of ED (VCAM-1, ICAM-1) and inflammation (IL-6) were associated with ME/CFS status (Fig. 4h).

Furthermore, associations between circulating biomarkers and RVA parameters were examined within PCS patients. After correction for multiple testing, AVR was inversely associated with CRP (ρ = -0.27), IL-6 (ρ = -0.27), transferrin (ρ = -0.34), and positively with transferrin saturation (ρ = 0.28), while CRAE was inversely associated with VCAM-1 (ρ = -0.33) (Fig. 4i).

## Discussion

This observational case-control study is the first to perform an in-depth assessment of retinal microvascular alterations using RVA as a surrogate of persistent ED across a cohort of NI individuals, recovered participants, and patients with PCS. We demonstrate that PCS patients exhibit markedly reduced venular FID, narrower retinal arterioles and a lower AVR compared with NI and recovered individuals. Higher symptom burden, particularly neurocognitive symptoms, was associated with retinal microvascular remodeling, as indicated by a lower AVR. These findings replicate and expand upon our previously published pilot study, in which comparable reductions were observed in a smaller PCS cohort compared to NI [52].

A key unresolved question was whether ED resolves during recovery or persists in association with post-viral symptomatology. Our analysis demonstrated lower vFID in PCS patients compared with recovered. These differences were independent of CV risk factors and remained evident after strict age-weighted analyses, supporting an association between persistent ED and ongoing post-viral syndromes. Reduced vFID provides functional evidence of impaired NV coupling and diminished endothelial-dependent vasodilatation. Retinal FID largely depends on NO release and glia cell-regulated NV coupling [29].

In individuals recovering from an acute infection, especially in those with pre-existing CV risk or severe acute illness longitudinal studies demonstrate persistent ED, by means of impaired endothelial glycocalyx, reduced NO bioavailability and vascular function [23, 53–56]. In contrast, recovered patients without relevant comorbidities generally show only minimal or transient ED, with biomarker levels returning toward baseline over time [57–61].

Our RVA findings suggest that patients with PCS fail to adequately restore endothelial integrity, potentially due to unresolved chronic inflammation and associated endothelial senescence, as reflected by persistently elevated IL-6, acute-phase proteins, and endothelial activation markers such as ICAM-1 and VCAM-1 in our cohort, which is consistent with previous reports [18, 62, 63]. Studies in PCS report gliosis and blood-brain barrier (BBB) disruption, and sustained neuroinflammation has been linked to persistent cognitive and affective symptoms in these patients [64, 65]. Additionally, the potent vasoconstrictor endothelin-1 (ET-1), which induces neuronal apoptosis and retinal ganglion cell loss, has been shown to be elevated in PCS patients and to correlate with ongoing symptom burden. Together, these findings support NV unit dysfunction in PCS and provide a plausible explanation for the observed reduction in vFID [23, 66, 67].

Regarding SVA parameters, we observed lower AVR and CRAE in PCS compared with both recovered and NI individuals, indicating persistent microvascular remodeling and impaired retinal autoregulation. Retinal calibers depend on age, diabetes, BMI, and hypertension [48]. The association between lower AVR and PCS was attenuated after adjustment for confounders and may partly reflect the higher CV risk profile of PCS patients compared with recovered. Lower AVR and CRAE reflect a relatively chronic vascular phenotype shaped by cumulative CV risk exposure and could partly represent pre-existing microvascular vulnerability associated with increased susceptibility to persistent post-infectious symptoms [68]. Altered SVA parameters in general have been consistently linked to CV risk in landmark studies [35, 69]. At the same time, substantial epidemiological evidence demonstrates that SARS-CoV-2 infection is associated with persistently increased long-term CV and cerebrovascular risk [20–22, 70]. In this context, reduced AVR and narrower retinal arterioles in PCS may reflect an interplay between baseline vascular vulnerability and sustained post-infectious microvascular remodeling beyond the acute phase of infection. Importantly, the association between lower AVR and greater PCS symptom burden suggests that these retinal microvascular alterations are not solely attributable to baseline CV risk factors but are also linked to PCS severity. In line with this, individuals with predominantly ongoing neurocognitive symptoms showed a lower AVR than recovered individuals or PCS patients without these symptoms. Given the established associations of lower AVR and arteriolar narrowing with cerebral small vessel disease, neuroinflammation, and cognitive impairment, reduced AVR and CRAE observed in PCS may provide evidence of persistent microvascular injury affecting NV integrity and offer a plausible mechanistic link to ongoing neurocognitive symptoms [71–74]. At a median disease duration of 13 months in our cohort, the persistence of these microvascular alterations suggests that RVA may provide a non-invasive window into sustained microvascular injury and potentially increased CV risk in patients with PCS.

Early recognition of the clinical and biological overlap between ME/CFS and PCS has shifted research toward a unified post-infectious disease framework [13, 63]. In our cohort, 61% of PCS patients met ME/CFS criteria and exhibited greater symptom burden and functional impairment. Across previously infected individuals, retinal ED followed a continuous gradient, with progressively lower vFID, CRAE, and AVR values corresponding to increasing clinical severity and the most pronounced alterations in PCS patients fulfilling ME/CFS criteria.

After age adjustment, IL-6, ICAM-1, and VCAM-1 remained elevated in ME/CFS and showed a similar pattern in PCS, indicating a shared inflammatory-endothelial activation axis across the post-infectious spectrum, with ME/CFS representing a more severe or biologically enriched phenotype [16, 66, 75]. AVR alone provided modest discrimination between ME/CFS and non-ME/CFS patients. However, its combination with routine laboratory parameters improved classification performance, reaching an AUC of 0.79, comparable to a previously reported diagnostic cell-based assay [76, 77]. Lower AVR correlated with higher IL-6 and CRP, linking retinal microvascular remodeling to systemic inflammation. Both markers have been associated with persistent neurocognitive symptoms in PCS, and IL-6-mediated neuroinflammation may provide a plausible mechanistic link to reduced AVR [78–82]. Beyond its inflammatory role, IL-6 is a key regulator of hepcidin and iron homeostasis [83, 84]. AVR was associated with transferrin levels and transferrin saturation, suggesting that retinal microvascular alterations in PCS may co-occur with an inflammatory-iron axis rather than isolated iron deficiency. Similar patterns of iron dysregulation and inflammatory stress erythropoiesis have been reported following COVID-19 and linked to long-term PCS outcomes [12, 83].

From a clinical and translational perspective, the non-invasive, repeatable, and relatively low-cost nature of RVA makes it well suited for longitudinal assessment in patients with persistent post-viral symptoms [85, 86]. RVA may complement symptom-based evaluation and routine laboratory testing by providing an objective, microvascular readout, particularly in patients with prominent neurocognitive impairment or ME/CFS features.

### Limitations

Several limitations warrant consideration. First, this is a cross-sectional, single-center cohort study; therefore, the observed associations, while biologically plausible, should not be interpreted as causal. The present findings are exploratory and hypothesis-generating and require validation in larger, independent and prospective cohorts. Second, despite adjustment for potential confounders, the recovered cohort was younger than the PCS group, which may have influenced retinal microvascular parameters. In addition, we cannot fully exclude that a subset of recovered individuals may still exhibit residual or transient ED, given that participants were assessed within > 3 months after acute infection. However, time since infection did not differ significantly between PCS and recovered individuals. Notably, altered RVA measures were also observed when PCS patients were compared with the NI cohort, which was older and had a higher burden of comorbidities, supporting the robustness of the findings. Finally, the cross-sectional design precludes conclusions regarding whether lower AVR and narrower CRAE represent post-infectious microvascular remodeling or a pre-existing vascular phenotype associated with increased susceptibility to PCS. Prospective longitudinal studies integrating RVA with adjudicated CV outcomes are required to determine whether retinal microvascular measures can identify a high-risk PCS subpopulation that may benefit from intensified CV monitoring and preventive strategies.

## Conclusions

This study demonstrates that patients with PCS exhibit persistent functional and structural alterations of the retinal microvasculature, indicating ongoing endothelial and NV dysfunction. Retinal ED is most pronounced in patients with higher symptom burden and in those fulfilling ME/CFS criteria, and is accompanied by sustained inflammatory and endothelial activation, as reflected by elevated IL-6, ICAM-1, and VCAM-1. The observed associations between retinal microvascular parameters, systemic inflammation, and iron homeostasis suggest that microvascular remodeling in PCS reflects a broader, inflammation-driven vascular phenotype rather than isolated CV risk. Together, these findings support the utility of RVA as a non-invasive window into post-infectious ED and highlight its potential role in biological stratification and risk assessment in patients with persistent symptoms. Prospective longitudinal studies are warranted to determine the prognostic value of retinal microvascular measures and their relevance for long-term CV outcomes in this population.

## Supplementary Information

Below is the link to the electronic supplementary material.

Supplementary Material 1: Additional file 1: Fig. S1 and Tables S1 to S11.

## Data Availability

De-identified participant-level data supporting the findings of this study are not publicly available due to ethical and data protection restrictions. Data are available from the corresponding authors upon reasonable request and subject to approval of a data access agreement and the relevant ethics requirements.

## Abbreviations

aFID: Arteriolar flicker–induced dilation
ANOVA: Analysis of variance
ATO: Average treatment effect in the overlap population
AUC: Area under the curve
AVR: Arteriolar–to–venular ratio
BBB: Blood–brain barrier
BMI: Body mass index
CCL: 5–CC–motif Chemokine–Ligand–5
CCC: Canadian Consensus Criteria
CI: Confidence interval
CK: Creatine kinase
CRAE: Central retinal arteriolar equivalent
CRP: C–reactive protein
CRVE: Central retinal venular equivalent
CV: Cardiovascular
C19: YRS–COVID–19 Yorkshire Rehabilitation Scale
CXCL: 10 (IP–10)–C–X–C motif chemokine 10 (Interferon gamma–induced protein–10)
DVA: Dynamic retinal vessel analysis
ED: Endothelial dysfunction
EQ: 5D–5 L–EuroQol five–dimension five–level questionnaire
ET: 1–Endothelin–1
FID: Flicker–induced dilation
FSS: Fatigue Severity Scale
GAD: 7–Generalized Anxiety Disorder–7
ICAM: 1–Intercellular adhesion molecule–1
IgG: Immunoglobulin G
IL: 6–Interleukin–6
IQR: Interquartile range
ME/CFS: Myalgic encephalomyelitis/chronic fatigue syndrome
MCP: 1–Monocyte chemoattractant protein–1
MRI: Magnetic resonance imaging
MuCOV: Munich COVID History Questionnaire
NI: Never infected
NO: Nitric oxide
NV: Neurovascular
PCS: Post–COVID–19 syndrome
PEM: Post–exertional malaise
PHQ: 9–Patient Health Questionnaire–9
PROMs: Patient–reported outcome measures
QC: Quality control
ROC: Receiver operating characteristic
RVA: Retinal vessel analysis
SD: Standard deviation
Std.β: Standardized beta coefficient
STROBE: Strengthening the Reporting of Observational Studies in Epidemiology
SVA: Static retinal vessel analysis
VCAM: 1–Vascular cell adhesion molecule–1
VEGF: Vascular endothelial growth factor
vFID: Venular flicker–induced dilation
WHO: World Health Organization
