# Supplementary material for: Microvascular remodeling and endothelial dysfunction across the post-COVID-19 spectrum: a prospective observational case-control study
Source: BMC Med. 2026 Aug 19;24:456. doi: 10.1186/s12916-026-05144-9 (PMC13495223; doi:10.1186/s12916-026-05144-9)
Supplement: Supplementary file 1 [file 12916_2026_5144_MOESM1_ESM.docx]

Additional File 1: Microvascular Remodeling and Endothelial Dysfunction Across the Post-COVID-19 Spectrum: A Prospective Observational Case-Control Study

Timon Wallraven^1*^, Roman Günthner^1^, Isabelle Lethen^1^, Andrea Ribeiro^1^, Maciej Lech^2^, Frederike Cosima Oertel^3,4,5^, Lukas Gabriel Reeß^3^, Bernhard Haller^6^, Lukas Streese^7,8^, Henner Hanssen^9^, Michael Basta-Wunderle^1#^ & Christoph Schmaderer^1,10#^

^1^TUM School of Medicine and Health, Department of Nephrology, TUM University Hospital,

Technical University of Munich, Munich, Germany

^2^Department of Medicine IV, LMU University Hospital, Munich, Germany

^3^Experimental and Clinical Research Center, Charité-Universitätsmedizin Berlin, Corporate Member of Freie Universität Berlin and Humboldt-Universität zu Berlin, and Max Delbrück Center for Molecular Medicine, Germany

^4^Einstein Center Digital Future Berlin, Berlin, Germany

^5^Department of Neurology, Charité-Universitätsmedizin Berlin, Corporate Member of Freie Universität Berlin and Humboldt-Universität zu Berlin, and Max Delbrück Center for Molecular Medicine, Germany

^6^TUM School of Medicine and Health, Institute for AI and Informatics in Medicine, TUM University Hospital, Technical University of Munich, Munich, Germany

^7^Faculty of Health Care, Therapeutic Sciences, Niederrhein University of Applied Sciences, Krefeld, Germany

^8^Department of Nephrology, Medical Faculty, University Hospital Düsseldorf, Heinrich-Heine-University Düsseldorf, Düsseldorf, Germany

^9^Department of Sport, Exercise and Health, Preventive Sports Medicine and Systems Physiology, University of Basel, Basel, Switzerland

^10^German Centre for Infection Research (DZIF), Partner Site Munich, Munich, Germany

#These authors contributed equally as senior authors.

Corresponding author:

T. Wallraven (timon.wallraven@mri.tum.de); Ismaninger Str. 22, 81675 München, Deutschland, 0049 89/4140 5231

**Additional File 1: Figure S1** Flowchart illustrating participant selection, exclusions, and analytic strategy. After screening and exclusions, 102 PCS and 102 recovered individuals were included. From the NI pool (n = 233), an age- and sex-matched NI cohort (n = 96) was derived for comparison with PCS. Recovered participants were analyzed in a 1:1 comparison framework with PCS. Sensitivity analyses using propensity-score overlap weighting (ATO) were performed to achieve covariate balance for age and sex, for PCS versus recovered comparisons. A subgroup of PCS patients fulfilling Canadian Consensus Criteria (CCC) for myalgic encephalomyelitis/chronic fatigue syndrome (ME/CFS) (n = 62) is indicated.

| **Covariate** | **Type** | **SMD (Unadjusted)** | **SMD (ATO-weighted)** |
| --- | --- | --- | --- |
| Propensity score | Distance | 0.826 | 0.002 |
| Age | Continuous | -0.715 | 0.000 |
| Male sex | Binary | 0.039 | 0.000 |
|  | | | |
|  | **Recovered** | **PCS** |  |
| **Sample size (unadjusted)** | 102 | 102 |  |
| **Effective sample size (ATO)** | 85.83 | 85.79 |  |

**Additional File 1: Table S1** Covariate balance before and after propensity-score overlap weighting targeting the average treatment effect in the overlap population (ATO) for the comparison between PCS and recovered individuals. Balance was assessed using standardized mean differences (SMD). After weighting, all covariates were well balanced (SMD < 0.01). Effective sample size (ESS) reflects the weighted pseudo-population.

| **Dependent variable ME/CFS** | | | |
| --- | --- | --- | --- |
| *Predictors* | *Odds Ratios* | *CI* | *p* |
| Transferrin | 0.987 | 0.976 – 0.997 | **0.013*** |
| IL-6 | 1.160 | 1.039 – 1.373 | **0.026*** |
| CK | 0.998 | 0.992 – 1.001 | 0.412 |
| AVR* | 0.338 | 0.170 – 0.622 | **0.001**** |
| Neutrophils | 1.061 | 1.015 – 1.113 | **0.012*** |
| ICAM-1 | 1.001 | 1.000 – 1.001 | **0.045*** |
| Observations | 165 | | |
| R^2^ Tjur | 0.235 | | |

**Additional File 1: Table S2** Multivariable logistic regression with ME/CFS status as the dependent variable. Results are shown as odds ratios (OR) with 95% confidence intervals (CI) and p-values. The model included arteriolar–venular ratio (AVR), interleukin-6 (IL-6), neutrophil percentage, creatine kinase (CK), intercellular adhesion molecule-1 (ICAM-1) and transferrin. *AVR scaled per 0.1 increase for interpretability. Model fit is summarized by Tjur’s R^2^. Statistical significance is indicated as: *p < 0.05; **p < 0.01; ***p < 0.001.

| **Laboratory value** | **Comparison** | **Model** | **Estimate (CI)** | **Std. β** | **p-value** |
| --- | --- | --- | --- | --- | --- |
| Hemoglobin | **NI vs PCS** | Age-adjusted | 0.75 (0.26 – 1.25) | 0.20 | **0.004**** |
|  | Recovered vs PCS | Age-adjusted | -0.34 (-0.75 – 0.07) | -0.12 | 0.103 |
| CRP | **NI vs PCS** | Age-adjusted | -1.04 (-0.76 – -1.93) | -0.41 | **<0.001***** |
|  | Recovered vs PCS | Age-adjusted | -0.05 (-0.46 – 0.38) | -0.02 | 0.682 |
| Leukocytes  (G/L) | **NI vs PCS** | Age-adjusted | -0.69 (-1.30 – -0.07) | -0.14 | **0.028*** |
|  | Recovered vs PCS | Age-adjusted | -0.24 (-0.74 – 0.25) | -0.07 | 0.335 |
| **Laboratory value** | **Comparison** | **Model** | **Delta (PCS – Recovered)** | **Std. β** | **p-value** |
| Neutrophils | **PCS vs Recovered** | ATO-weighted | 3.82 (0.94 – 6.69) | — | **0.010**** |
| Lymphocytes | PCS vs Recovered | ATO-weighted | -1.90 (-4.51 – 0.70) | — | 0.151 |
| Platelets | PCS vs Recovered | ATO-weighted | 13.8 (-5.38 – 33.0) | — | 0.158 |
| Ferritin  log | PCS vs Recovered | ATO-weighted | 0.21 (-0.10 – 0.52) | — | 0.187 |
| Transferrin | **PCS vs Recovered** | ATO-weighted | -15.5 (-26.7 – -4.28) | — | **0.007**** |
| LDL | PCS vs Recovered | ATO-weighted | 10.5 (-1.78 – 22.8) | — | 0.093 |
| IgG3 | PCS vs Recovered | ATO-weighted | -4.34 (-9.37 – 0.69) | — | 0.091 |
| CK  log | **PCS vs Recovered** | ATO-weighted | -0.41 (-0.61 – -0.21) | — | **<0.001***** |

**Additional File 1: Table S3** Associations between cohort status and laboratory parameters including hemoglobin, C-reactive protein (CRP), and leukocyte counts, estimated using age-adjusted linear regression models across NI, recovered, and PCS participants, with PCS as the reference category. In addition, group differences between PCS and recovered individuals for neutrophils, lymphocytes, platelets, ferritin, transferrin, LDL cholesterol, IgG3, and creatine kinase (CK) were re-estimated in an age- and sex-balanced pseudo-population using propensity score-based overlap weighting (ATO). Survey-weighted linear models were used for approximately normally distributed variables, while survey-weighted rank-based tests were applied for non-normally distributed variables (ferritin, IgG3, CK). Estimates are presented as β coefficients or mean differences with 95% confidence intervals. Standardized β coefficients are reported for regression models. Negative values indicate lower values in PCS compared with recovered individuals. *p < 0.05; **p < 0.01; ***p < 0.001.

| **Variable** | **Mean difference**  **(PCS - Recovered)** | **95% CI** | **p-value** |
| --- | --- | --- | --- |
| AVR | -0.018 | -0.04 to 0.00 | 0.056 |
| **CRAE (µm)** | **-4.87** | **-9.59 to -0.15** | **0.043*** |
| CRVE (µm) | -1.44 | -6.12 to 3.24 | 0.544 |
| **vFID (%)** | **-1.75** | **-2.52 to -0.970** | **<0.001***** |
| aFID (%) | -0.44 | -1.09 to 0.22 | 0.189 |

**Additional File 1: Table S4** Sensitivity analysis adjusting for age and sex using overlap weighting (ATO). Group differences in RVA parameters were re-estimated using survey-weighted linear models in an age- and sex-balanced pseudo-population. Negative values indicate lower values in PCS than in recovered individuals.*p < 0.05; ***p < 0.001.

| **Parameter** | **Recovered**  (n=45) | **PCS**  (n=45) | **p** |
| --- | --- | --- | --- |
| Age (years) | 36.6 ± 11.3 | 36.6 ± 11.3 | 1.000 |
| Female sex, n (%) | 36 (80.0%) | 36 (80.0%) | 1.000 |
| **CRAE (µm)** | **188.6 ± 14.3** | **180.7 ± 13.1** | **0.008**** |
| CRVE (µm) | 218.1 ± 14.3 | 217.1 ± 15.8 | 0.762 |
| **AVR** | **0.867 ± 0.065** | **0.832 ± 0.050** | **0.007**** |
| **vFID (%)** | **4.90 ± 2.93** | **3.45 ± 2.09** | **0.010*** |
| aFID (%) | 3.34 ± 2.60 | 2.72 ± 2.21 | 0.232 |

**Additional File 1: Table S5** Participants were exactly matched 1:1 on age and sex to minimize residual confounding between recovered and PCS groups. Values are shown as mean ± SD. Group comparisons were performed using Welch’s two-sample t-test. *p < 0.05; **p < 0.01; ***p < 0.001.

| **Dependent variable vFID** | | | | |
| --- | --- | --- | --- | --- |
| **Predictor** | **Estimate** | **95% CI** | **Standardized β** | **p** |
| **NI (vs PCS)** | 0.25 | −0.48 to 0.98 | 0.05 | 0.504 |
| **Recovered (vs PCS)** | **1.27** | **0.52 to 2.03** | **0.24** | **0.001**** |
| **Age** | 0.03 | 0.01 to 0.06 | 0.18 | **0.008**** |
| BMI | 0.07 | -0.01 to 0.15 | 0.12 | 0.064 |
| Arterial hypertension | -0.75 | −1.97 to 0.47 | −0.08 | 0.229 |
| Systolic BP | -0.01 | -0.03 to 0.01 | -0.08 | 0.227 |

| **Dependent variable CRAE** | | | | | |
| --- | --- | --- | --- | --- | --- |
| **Predictor** | **Estimate** | **95% CI** | **p** | **Standardized β** | **p** |
| **Never-Infected (vs PCS)** | **5.58** | **1.33 to 9.83** | **0.010*** | **0.17** | **0.010*** |
| Recovered (vs PCS) | 3.35 | -0.99 to 7.69 | 0.130 | 0.10 | 0.130 |
| Age | -0.32 | -0.46 to -0.18 | <0.001*** | -0.27 | <0.001*** |
| Arterial hypertension | -6.04 | -13.09 to 1.00 | 0.093 | -0.10 | 0.093 |
| BMI | 0.08 | -0.35 to 0.51 | 0.713 | 0.02 | 0.713 |
| Systolic BP | -0.20 | -0.31 to -0.09 | <0.001*** | -0.21 | <0.001*** |

| **Dependent variable AVR** | | | | | |
| --- | --- | --- | --- | --- | --- |
| **Predictor** | **Estimate** | **95% CI** | **p** | **Standardized β** | **p** |
| **Never-Infected (vs PCS)** | **0.03** | **0.01 to 0.05** | **0.001***** | **0.23** | **0.001***** |
| Recovered (vs PCS) | 0.01 | -0.00 to 0.03 | 0.139 | 0.10 | 0.139 |
| Age | -0.0003 | -0.0009 to 0.0003 | 0.348 | -0.06 | 0.348 |
| Arterial hypertension | -0.012 | -0.044 to 0.019 | 0.444 | -0.05 | 0.444 |
| BMI | -0.0018 | -0.0037 to 0.0002 | 0.073 | -0.11 | 0.073 |
| Systolic BP | -0.0005 | -0.0010 to 0.0000 | 0.053 | -0.12 | 0.053 |

**Additional File 1: Table S6** Multivariable linear regression models assessing associations between cohort status (NI and recovered, each compared with the reference group PCS) and RVA parameters, including vFID, CRAE and AVR. Adjusted models include age, arterial hypertension, body mass index (BMI), and systolic blood pressure (BP) prior to retinal vessel analysis. Estimates are presented as regression coefficients with 95% confidence intervals (CI). Standardized ß-coefficients are additionally presented. PCS served as the reference category in all models. Significance levels are indicated as *p < 0.05 , ** p < 0.01 and *** p < 0.001.

| **Symptom** | **AVR (Symptom Absent)** | **AVR (Symptom Present)** | **p-value** |
| --- | --- | --- | --- |
| Fatigue | 0.856 | 0.833 | **0.009**** |
| Concentration difficulties | 0.857 | 0.831 | **0.005**** |
| Headache | 0.856 | 0.829 | **0.005**** |
| Anxiety and/or sleep disturbance | 0.852 | 0.833 | **0.036*** |
| Balance/fine motor dysfunction | 0.851 | 0.829 | **0.026*** |
| Chest pain | 0.847 | 0.839 | 0.380 |
| Palpitations | 0.855 | 0.829 | **0.005**** |
| Shortness of breath | 0.849 | 0.831 | 0.085 |
| Cough | 0.848 | 0.831 | 0.230 |
| Reduced physical capacity (e.g., difficulty climbing stairs) | 0.855 | 0.835 | **0.027*** |
| Disturbed smell or taste | 0.847 | 0.835 | 0.440 |
| ENT symptoms (e.g., sore throat, runny nose, hoarseness) | 0.850 | 0.829 | **0.041*** |
| Hair loss / itching / skin rash | 0.850 | 0.822 | **0.008**** |
| Weight loss | 0.846 | 0.836 | 0.430 |
| Gastrointestinal symptoms (e.g., nausea, diarrhea) | 0.848 | 0.829 | **0.043** |
| General malaise / chills / flu-like symptoms | 0.853 | 0.828 | **0.010*** |

**Additional File 1: Table S7** Mean AVR in participants who did not report (“Symptom absent”) versus did report (“Symptom present”) each of the 16 symptoms included in the PCS symptom severity score. For each symptom, mean AVR values are shown for both groups and p-values from group comparisons. Symptoms are listed in the order included in the PCS symptom severity score and span neurological, cardiopulmonary, sensory, and systemic domains. Group comparisons were performed using Welch’s two-sample t-test or the Mann-Whitney U test, as appropriate. Statistical significance is indicated as *p < 0.05; **p < 0.01; ***p < 0.001.

| **Variable** | **PCS without ME/CFS N = 39** | **PCS with ME/CFS N = 62** | **p-value** |
| --- | --- | --- | --- |
| Age (years), mean (±SD) | 44.8 (±11.6) | 39.9 (±11.2) | **0.041*** |
| Sex (female), n(%) | 28 (72%) | 49 (79%) | 0.6 |
| BMI (kg/m^2^), mean (±SD) | 24.4 (±4.6) | 24.6 (±4.9) | 0.8 |
| Resting systolic BP before RVA^1^(mmHg), mean (±SD) | 121.9 (±18.8) | 121.7 (±15.6) | >0.9 |
| **Cardiovascular Risk Factors** | | | |
| Diabetes mellitus (I and II), n(%) | 1 (2.6%) | 0 (0%) | >0.9 |
| Arterial Hypertension, n(%) | 5 (13%) | 11 (18%) | 0.7 |
| Hypercholesterolemia, n(%) | 7 (18%) | 15 (24%) | 0.6 |
| Smoking history, n(%) | 4 (10%) | 6 (9.7%) | >0.9 |
| **PCS-related characteristics** | | | |
| C19-YRS*,* mean (±SD) | 28.3 (15.1) | 35.0 (12.7) | **0.023*** |
| PCS Severity Score*,* mean (±SD) | 32.0 (11.7) | 40.0 (8.6) | **<0.001***** |
| PHQ9*,* mean (±SD) | 8.9 (4.7) | 11.4 (3.9) | **0.008**** |
| GAD7*,* median (IQR) | 4.0 (2.0, 7.0) | 6.0 (3.0, 9.0) | 0.2 |
| FSS, median (IQR) | 5.4 (3.7, 6.4) | 6.3 (5.8, 6.7) | **<0.001***** |
| EQ5DL-Index, median (IQR) | 0.8 (0.6, 0.8) | 0.5 (0.3, 0.7) | **0.010*** |
| Duration of PCS, days, median (IQR) | 345.0 (218.0, 537.0) | 409.5 (316.5, 619.0) | 0.15 |
| Cumulative sick days, median (IQR) | 129.0 (0.0, 291.0) | 285.5 (106.0, 445.0) | **<0.001***** |
| Work loss during PCS, n(%) | 4 (10%) | 12 (20%) | 0.3 |
| **Acute SARS-CoV-2 infection** | | | |
| Number of infections*,* n(%) |  |  | 0.2 |
| 1 | 30 (77%) | 40 (65%) |  |
| 2 | 8 (21%) | 21 (34%) |  |
| 3 | 1 (2.6%) | 0 (0%) |  |
| 4 | 0 (0%) | 1 (1.6%) |  |
| Severity of acute infection*,* n(%)  (WHO progression scale) |  |  | 0.6 |
| 1 | 1 (2.6%) | 0 (0%) |  |
| 2 | 25 (64%) | 35 (56%) |  |
| 3 | 12 (31%) | 22 (35%) |  |
| 4 | 1 (2.6%) | 3 (4.8%) |  |
| 5 | 0 (0%) | 1 (1.6%) |  |
| 6 | 0 (0%) | 1 (1.6%) |  |
| **Lab parameters** | | | |
| Creatine Kinase (U/L), median IQR) | 91.0 (70.0, 111.0) | 71.0 (54.0, 92.0) | **0.009**** |
| **Comorbidities** | | | |
| Psychiatric Diagnosis preexisting*,* n(%) | 2 (5.3%) | 6 (9.7%) | 0.7 |
| Depressive diagnosis ongoing*,* n(%) | 9 (23%) | 10 (16%) | 0.5 |

**Additional File 1: Table S8** Baseline characteristics of PCS patients stratified by ME/CFS status according to the Canadian Consensus Criteria (CCC). One patient did not complete the CCC questionnaire and was therefore excluded from the analysis. Continuous variables are reported as mean ± SD or median (IQR), depending on distribution, and categorical variables as n (%). Group comparisons were performed using Welch’s two-sample t-test for normally distributed continuous variables, the Mann-Whitney U test for non-normally distributed continuous variables, and Pearson’s χ² test for categorical variables, as appropriate. Abbreviations: BMI, body mass index; Resting systolic BP before RVA, systolic blood pressure before retinal vessel analysis; C19-YRS, COVID-19 Yorkshire Rehabilitation Scale; PHQ-9, Patient Health Questionnaire-9; GAD-7, Generalized Anxiety Disorder-7; FSS, Fatigue Severity Scale; EQ-5D-5L, EuroQol 5-Dimension 5-Level index; ME/CFS, myalgic encephalomyelitis/chronic fatigue syndrome. Statistical significance is indicated as *p < 0.05; **p < 0.01; ***p < 0.001.

| **vFID as the dependent variable** | | |
| --- | --- | --- |
| *Predictors* | *Estimates*  *(Std. ß)* | *p* |
| NI | 0.11 (0.02) | 0.792 |
| PCS without ME/CFS | −0.28 (−0.04) | 0.600 |
| **Recovered** | **1.19 (0.22)** | **0.006**** |
| Age | 0.03 (0.16) | 0.016* |
| Sex [male] | −0.47 (−0.08) | 0.207 |
| BMI | 0.07 (0.12) | 0.066 |
| Resting systolic BP before RVA^1^ | −0.01 (−0.04) | 0.556 |
| Arterial hypertension | −0.77 (−0.08) | 0.218 |

| **CRAE as the dependent variable** | | |
| --- | --- | --- |
| *Predictors* | *Estimates*  *(Std. ß)* | *p* |
| **NI** | **6.29 (0.19)** | **0.013** |
| PCS without ME/CFS | 1.53 (0.03) | 0.615 |
| Recovered | 3.94 (0.12) | 0.114 |
| Age | -0.32 | **<0.001***** |
| Sex [male] | -1.21 | 0.890 |
| BMI | 0.08 | 0.709 |
| Resting systolic BP before RVA^1^ | -0.21 | **<0.001***** |
| Arterial hypertension | -5.94 | 0.100 |

| **AVR as the dependent variable** | | |
| --- | --- | --- |
| *Predictors* | *Estimates*  *(Std. ß)* | *p* |
| **NI** | **0.04 (0.31)** | **<0.001***** |
| PCS without ME/CFS | 0.03 (0.14) | **0.052** |
| **Recovered** | **0.02 (0.17)** | **0.029*** |
| Age | -0.00 | 0.422 |
| Sex [male] | 0.01 | 0.130 |
| BMI | -0.00 | 0.085 |
| Resting systolic BP before RVA^1^ | -0.00 | **0.018*** |
| Arterial hypertension | -0.01 | 0.513 |

**Additional File 1: Table S9** Multivariable regression analyses examining associations between cohort subgroups never-infected (NI), recovered individuals, and PCS patients without ME/CFS and RVA parameters vFID, CRAE and AVR. All models were adjusted for age, sex, body mass index (BMI), systolic blood pressure prior to retinal vessel analysis (Resting systolic BP before RVA), and arterial hypertension. Standardized (Std.) ß-coefficients are presented in brackets. The reference group is PCS patients fulfilling ME/CFS criteria. Statistical significance is indicated as ***p *<* 0.05, **p < 0.01, **and ******* p ***<* 0.001.**

| **Variable** | **OR (95% CI)** | **p-value** | **FDR-adjusted p** | **AUC (95% CI)** | **Biological Domain** |
| --- | --- | --- | --- | --- | --- |
| **Creatine kinase** | 1.00 (0.99–1.00) | 0.128 | 0.202 | **0.73 (0.65–0.80)** | Muscle metabolism |
| **IL-6** | 1.26 (1.09–1.51) | 0.007 | 0.035* | 0.70 (0.61–0.78) | Inflammation |
| CRP | 1.27 (0.45–3.36) | 0.616 | 0.704 | 0.67 (0.57–0.76) | Inflammation |
| VCAM-1 | 1.000 (1.000–1.000) | 0.033 | 0.065 | 0.64 (0.56–0.73) | Endothelial activation |
| **ICAM-1** | 1.001 (1.000–1.001) | 0.006 | 0.035* | 0.63 (0.55–0.72) | Endothelial activation |
| **AVR*** | 0.50 (0.30–0.80) | 0.005 | 0.035* | 0.61 (0.53–0.70) | Retinal microcirculation |
| **Transferrin** | 0.99 (0.98–1.00) | 0.011 | 0.042* | 0.61 (0.52–0.69) | Iron metabolism |
| **Neutrophils** | 1.05 (1.01–1.09) | 0.017 | 0.056 | 0.60 (0.51–0.69) | Inflammation |
| aFID | 0.84 (0.71–0.98) | 0.030 | 0.065 | 0.61 (0.53–0.70) | Retinal microcirculation |
| CRAE | 0.98 (0.96–1.00) | 0.030 | 0.065 | 0.59 (0.51–0.68) | Retinal microcirculation |
| vFID | 0.91 (0.81–1.03) | 0.139 | 0.202 | 0.57 (0.49–0.65) | Retinal microcirculation |

**Additional File 1: Table S10** Univariable logistic regression and receiver operating characteristic (ROC) analyses for discrimination of ME/CFS among previously infected individuals. Odds ratios (ORs) with 95% confidence intervals (CI) describe the association between each variable and ME/CFS status. P-values are reported as both unadjusted and adjusted for multiple testing using the Benjamini–Hochberg false discovery rate (FDR) procedure. Discriminatory performance is reported as area under the ROC curve (AUC) and the CI calculated using DeLongs´method. For each variable the biological domain is shown. Abbreviations: IL-6, interleukin-6; CRP, C-reactive protein; ICAM-1, intercellular adhesion molecule-1; VCAM-1, vascular cell adhesion molecule-1; IL6, ICAM-1,VCAM-1, all n=190, * AVR scaled per 0.1 increase. Statistical significance is indicated as ***p *<* 0.05, **p < 0.01, **and ******* p ***<* 0.001.**

| **Biomarker** | **Mean difference (PCS - Recovered)** | **95% CI** | **p-value** |
| --- | --- | --- | --- |
| **ICAM-1 (pg/mL)** | **377** | 193 to 561 | **<0.001***** |
| **VCAM-1 (pg/mL)** | **4409** | 1547 to 7271 | **0.003**** |
| MCP-1 (pg/mL) | 4.5 | -3.0 to 12.0 | 0.235 |
| IL-6 (pg/mL) | 0.34 | -0.78 to 1.46 | 0.548 |
| VEGF (pg/mL) | -0.35 | -6.77 to 6.07 | 0.915 |

**Additional File 1: Table S11** Estimated mean differences (delta) in circulating biomarker levels between PCS and recovered individuals derived from overlap weighting targeting the average treatment effect in the overlap population (ATO). Results are presented as weighted mean differences (PCS – recovered) with 95% confidence intervals (CI) and corresponding p-values. Statistical significance is indicated as: *p < 0.05; **p < 0.01; ***p < 0.001.
